# *‘For the poor, sleep is leisure’*: understanding perceptions, barriers and motivators to mosquito net care and repair in southern Tanzania

**DOI:** 10.1186/s12936-018-2528-6

**Published:** 2018-10-22

**Authors:** Zawadi M. Mboma, Angel Dillip, Karen Kramer, Hannah Koenker, George Greer, Lena M. Lorenz

**Affiliations:** 1 Ifakara Health Institute, Dar-es-Salaam, Tanzania; 2 London School of Hygiene and Tropical Medicine, London, UK; 3 National Malaria Control Programme, Dar-es-Salaam, Tanzania; 4 Swiss Tropical and Public Health Institute, Basel, Switzerland; 5 University of Basel, Petersplatz 1, 4003 Basel, Switzerland; 6 PMI VectorWorks, Johns Hopkins Center for Communication Programs, Baltimore, MD USA; 7 U.S. President’s Malaria Initiative, U.S. Agency for International Development, Dar es Salaam, Tanzania

**Keywords:** Long-lasting insecticidal nets (LLINs), Mosquito net, Net care, Net repair, Malaria Tanzania, Health Belief Model

## Abstract

**Background:**

The rate of physical deterioration of long-lasting insecticidal nets (LLINs) varies by household practices, net brand and environment. One way to sustain the protection provided by LLINs against malaria is through day-to-day care, and repairing holes as and when they occur. To ensure LLIN coverage is high between mass campaigns and, as international donor funds decrease, personal responsibility to maintain nets in good condition is becoming more important. This study aimed to understand local barriers and motivators to net care and repair in southern Tanzania in a community that receives free LLINs through a school-based distribution mechanism.

**Methods:**

Qualitative research methods were applied in a rural and peri-urban village in Ruangwa district. Focus group discussions (FGDs) were conducted for five groups of 8–12 participants; (1) key informants, (2) young men (18–24 years old), (3) women (> 18 years) with children under the age of five, (4) older men (> 25 years), and (5) older women with or without children (> 25 years). In each village, five men, five women with or without children, and five women with children under the age of five were recruited for in-depth interviews (IDIs). After each IDI and FGD with women with young children, participants were guided through a participatory activity. The study also counted the number and size of holes in nets currently used by IDI participants to determine their physical degradation status.

**Results:**

A general willingness to care and repair mosquito nets was observed in Ruangwa district for the love of a good night’s sleep free of mosquito bites or noises. Net care was preferred over repair, especially among women who were the primary caretakers. The main motivation to look after nets was protection against mosquito bites and malaria. Washing nets occurred as frequently as every other week in some households to ensure cleanliness, which prevented other dirt-related problems such as sneezing and headaches. Barriers to net care included care not being a priority in the day-to-day activities and lack of net retreatment kits. Net repair was reported to be a temporary measure and necessary as soon as a hole was identified. However, during the net assessment and participatory activity, it became clear that people did not actually repair smaller holes. Protection against mosquitoes, malaria and cost saving from replacing nets were identified as motivators for net repair. Barriers to net repair included it not being a priority to repair holes that could be tucked under the mattress and lack of knowledge on when to repair nets.

**Conclusion:**

In Ruangwa, net care was defined as overall net maintenance, such as cleanliness, and not directly associated with the prevention of damage as reported in other studies. Net repair was reported as a temporary measure before the acquisition of a new net, hence not a priority in a busy household. Inconsistencies were observed between reported intentions to repair mosquito nets and current net condition. Targeted education through health facilities and community change agents are potential means to overcome barriers to net care and repair.

## Background

The Government of Tanzania has made considerable effort in achieving universal coverage for its population with Long-Lasting Insecticidal Nets (LLINs) through a number of continuous and keep-up distribution mechanisms [1–3]. The physical deterioration of the net, while inevitable with time, varies by product type, household practices (e.g. use, washing) and environment (e.g. type of sleeping space) [4–8]. One of the ways to sustain the protection provided by LLINs is through personal responsibility of households to care for LLINs day-by-day [9]. Extending the lifespan of LLINs is important to reduce the frequency of net replacements and maintain high access to LLINs between distributions, to ensure continuing health gains from the use of nets [5].

The World Health Organization Pesticide Evaluation Scheme (WHOPES), now replaced by the Prequalification Team (PQT), recommends that LLINs remain effective after 20 standard washes and last 3 years under field conditions [10]. Manufacturers instruct specific care practices to prolong the useful life of the LLIN, such as hanging the net low enough to touch the ground or tucking underneath the mattress, washing gently with soap and water but not bleach, drying nets in the shade and avoiding direct sunlight, keeping net away from direct flames and repairing holes as soon as possible [11]. However, it is unclear how many households receive their nets with the packaging or if those who receive the instructions on the packaging understand and practice them.

Net care (i.e. hanging of net, daily storage/tying up net over sleeping space, washing, drying, seasonal storage) and repair (i.e. sewing, knotting, patching) practices are similar across communities, but vary in priority between households [12–14]. In Senegal [13], Nigeria [14] and Mali [12], net care was preferred and more common than repair. In Uganda [5], nets perceived too torn were most likely to be repurposed for alternative uses around the house rather than repaired. In urban Dar-es-Salaam, requesting users to reduce washing frequency to maintain enough insecticide on nets was deemed impractical [15]. This variation in priority of performing net care and repair practices emphasizes the need to integrate local and culturally-fitting messaging with ongoing malaria interventions rather than promoting blanket universal recommendations across all endemic countries [16].

**Fig. 1:**
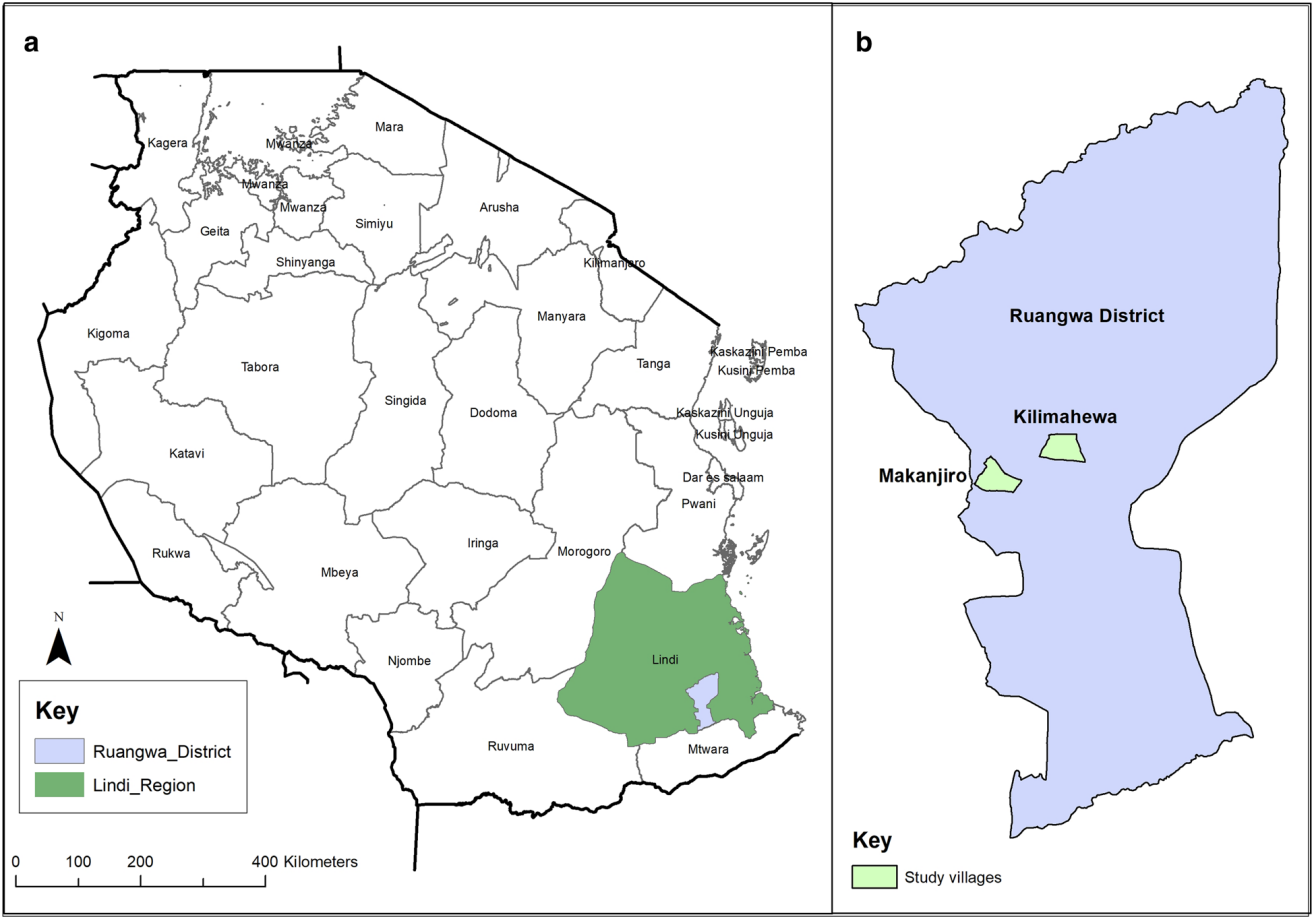
A map of the study sites: **a** The map of Tanzania with reference to the study region, **b** study villages in Ruangwa district

This study was conducted in southern Tanzania (Ruangwa district, Lindi region; Fig. 1) in 2016 after the third round of continuous LLIN distribution through the School Net Programme (SNP) conducted in 2015. Malaria prevalence in children under five in the Lindi Region remains high at 17.4% as per the 2015–2016 national health survey [17]. Starting in 2013, the SNP was introduced as a pilot “keep-up” strategy to supplement mass distribution campaigns as a means to maintain universal coverage of LLINs prior to its national roll-out [18, 19]. The programme distributes LLINs each year to school-going children in alternating classes (primary classes 1, 2, 3, 4, 5 and 7, secondary classes/forms 2 and 4) [18, 20, 21]. Ninety-eight percent of all registered students and teachers in Ruangwa district received LLINs through the SNP programme [20]. Generally in Lindi region, ownership of at least one LLIN was 70% while ownership of at least one LLIN for every two people who slept in the household the night prior to the survey was 47% according to the 2015–2016 National Health Survey [17]. Specifically, monitoring of SNP rounds 1 and 2 recorded ownership of at least one LLIN in all the SNP participating regions (Ruvuma, Lindi and Mtwara) to be 76% and 79%, respectively [21]. The analysis of the third mosquito net distribution is still ongoing. The SNP also promoted sharing of surplus nets with neighbours who did not own mosquito nets. Long-lasting insecticidal nets were to remain available to pregnant women and infants during antenatal and immunization visits at their attending health facility through the Tanzania National Voucher Scheme (TNVS) [18, 19]. Unfortunately, the TNVS was discontinued in 2014 and a replacement system (free nets during antenatal and immunization visits (ANC/EPI)) was not implemented until June 2016 (pers. Comm. Ikupa Akim, National Malaria Control Programme) [21, 22]. Alternative sources of mosquito nets (treated and untreated) are through the commercial sector (local market, kiosks) for those without school-going children.

**Fig. 2:**
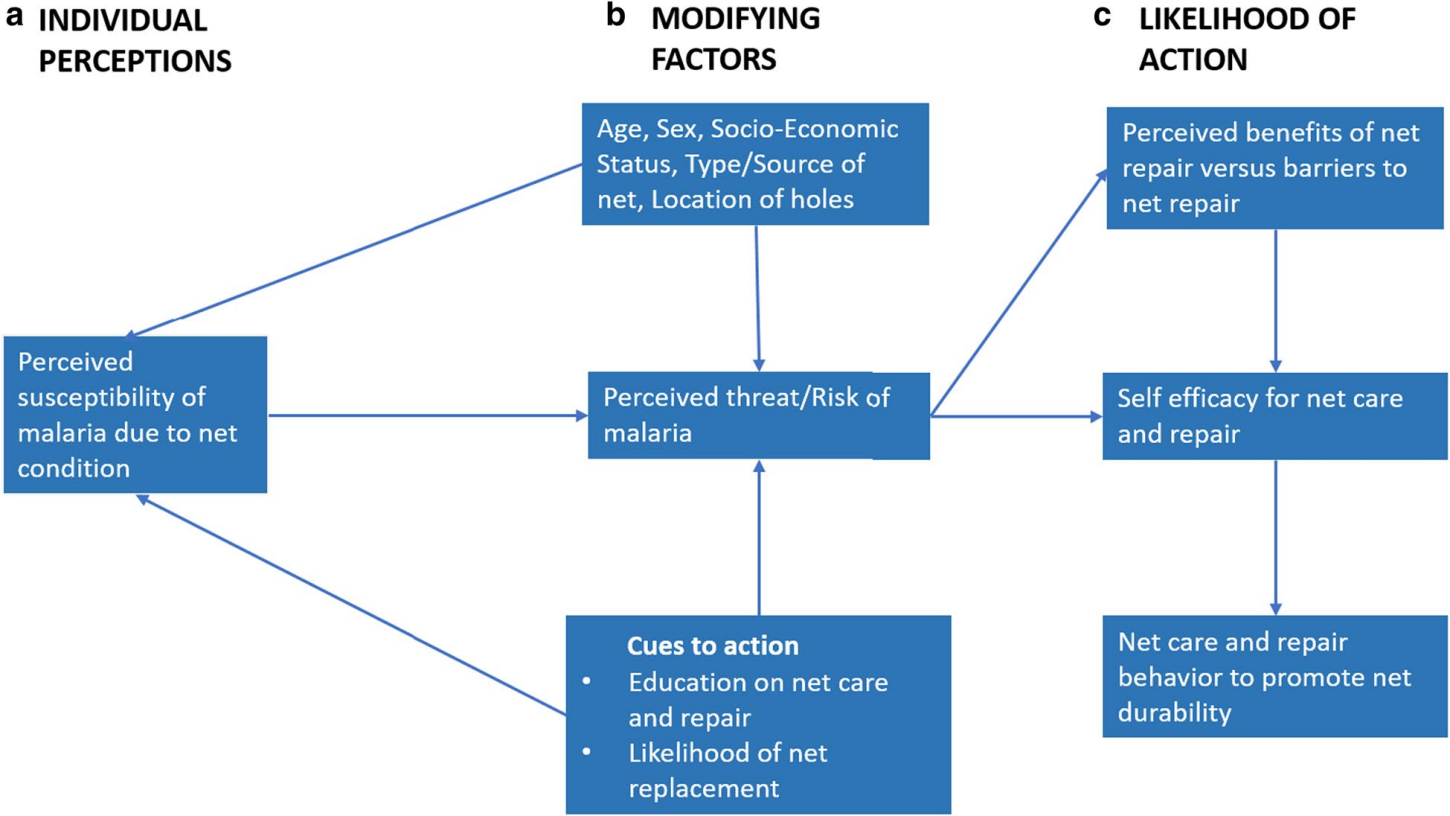
A conceptual model for net care and repair behaviours according to the Health Belief Model [23]. The model assumes **a** individual perceptions that malaria is a major public health threat; **b** modifying factors identify users as capable to perform day-to-day care and repair activities; and **c** likelihood of action to maintain nets as a means to protect themselves against malaria

The objective of this study was to explore local perceptions and practices of net care and repair in a community that continuously receives LLINs. Specifically, actions associated with different levels of net damage, motivators and barriers associated with net care and repair, and perceptions on how to overcome those reported barriers were assessed. The study approach was based on the Health Belief Model (HBM) [23], which has been useful to explain and predict human-disease interactions in previous studies [13, 24]. The model assumes that individuals will (a) opt to care for and repair their LLINs because of their perception that malaria is a major threat to their health (perceived severity and susceptibility), (b) identify themselves as capable to perform day-to-day care and repair activities (self-efficacy) based on modifying factors such as personal and net characteristics and external and internal cues to action, and (c) maintain nets as a means to protect themselves against malaria (perceived benefits increasing likelihood of action) (Fig. 2).

Understanding variations in local perceptions, motivators and barriers to net care and repair is key for the National Malaria Control Programme (NMCP) to optimize cost-effectiveness with fewer net replacements through suitable Behaviour-Change Communication (BCC). Exposure to effective BCC about net care and repair has been observed to improve overall net condition [25, 26]. However, repairs alone were not found sufficient to improve physical condition [25, 26], leading to the U.S President’s Malaria Initiative (PMI) to change their policy to support only net care initiatives promoting BCC that protects nets from damage and improve net use [27]. Reinforcing Tanzania’s BCC strategy to include relatable positive messages could inspire appropriate net care actions. The study expected participants to put high value on net care and repair to maintain intact nets as a valuable commodity that protects them against malaria, which they see as a major threat to their health (Fig. 2).

## Methods

### Study site

The study was conducted in Makanjiro (rural) and Kilimahewa (peri-urban) villages in Ruangwa District, Lindi Region (Fig. 1). Ruangwa District was one of two districts in Southern Zone enrolled in the population arm of the Sentinel Panel of Districts (SPD), Sample Vital registration with Verbal Autopsy (SAVVY) project based at the Ifakara Health Institute (IHI) [28]. Makanjiro and Kilimahewa villages were randomly selected from a pool of 15 villages enrolled in the SAVVY project. The primary malaria vectors in Tanzania are *Anopheles gambiae* sensu stricto*, Anopheles funestus* (both vectors indoor resting) and *Anopheles arabiensis* (outdoor resting) [19, 29–32]. Lindi region has one major rainy season per year (March–May) at the end of which peak malaria transmission occurs [19].

### Ethical approval and consent to participate

Ethical approval was obtained from the Ifakara Health Institute (Ref: IHI/IRB/No: 015-2016), and the National Institute of Medical Research, Tanzania (Ref: NIMR/HQ/R.8a/Vol. IX/2193). The study was only administered to participants above 18 years of age upon written informed consent.

### Data collection

Data was collected through a mix of qualitative research methods, namely Focus Group Discussions (FGDs), In-Depth Interviews (IDIs) and a Participatory Activity (PA). Study participants were selected purposively with the assistance of village leaders. Participants were eligible if they were above 18 years of age, had lived in the village for a minimum of 12 months, and owned at least one LLIN in their household.

In 2016, a pilot study was conducted in Pemba Mnazi (rural Dar-es-Salaam) to ensure research tools were locally appropriate. All FGDs and IDIs were conducted in Kiswahili language and audio-recorded with hand-held digital devices. In addition, notes were taken during each interview. Interviews were guided by a topic guide containing a priori themes identified through literature and based on the theoretical framework of the HBM model (Fig. 2). Participants were encouraged to narrate their day-to-day activities regarding care and repair of LLINs. The topic guide was used to probe where necessary. The sample size of 30 IDIs and 10 FGDs were determined by reviewing similar studies [5, 13, 14] to capture variation of responses from different participant groups. Response saturation [33] was reached after three FGDs and five IDIs, but sampling was continued to ensure emerging themes were not missed.

### Structured participant questionnaire

Prior to the start of any FGD or IDI, researchers administered a simple structured questionnaire to collect non-identifying socio-demographic information about each participant, including sex, age, education, number of children, participation in the SNP and exposure to BCC messaging in the past 6 months.

### Focus group discussions

Five FGDs were conducted in each village. Four FGDs were conducted with community members and one with key village informants (i.e. religious, traditional/village leaders, and influential people). The community members were split into four groups of 8–12 participants each. Focus Group Discussions were conducted separately for young men (18–24 years old), women (> 18 years) with children under the age of five, older men (> 25 years), and older women with or without children (> 25 years old).

### In-depth interviews

In each of the two villages, five men, five women with or without children, and five women with children under the age of five were recruited for IDIs. In-Depth Interviews were conducted primarily at the study participant’s home or space of comfort with minimal distraction from children and neighbours to provide a confidential environment for them to discuss in detail their attitudes and actions towards net care and repair.

### Participatory activity and mosquito net assessment

After each IDI and the FGD with women with children under the age of five, participants were guided through a participatory activity (PA). Study participants were shown individually labelled nets with different levels of damage and repair (Table 1) and were asked to decide between four actions for each net: (1) do nothing and continue to use; (2) repair and continue to use; (3) no longer use net but use it for something else in the household; or (4) no longer use it and discard the net. The level of damage and evidence of repair presented during the PA was to mimic observations from other field studies [34, 35]. Study participants were asked to make two choices for each net to explore current actions and understanding of net care and repair with social norms and discuss the reasons for their choices; (1) what they *would* do; and (2) what they think they *should* do.

**Table 1:** Responses for action on nets with different damage and repair attributes presented in the participatory activity

Net ID	Number of holes	Hole sizes^a^	Hole location^b^	Repair^c^	Category^d^	Common “*would do*” response	Common “*should do*” response
1	1	“Size 2”	Bottom	No	Good	Repair and continue to use	Repair and continue to use
2	1	“Size 2”	Roof	No	Good	Repair and continue to use	Repair and continue to use
3	18	15 × “Size 1”, 3 × “Size 2”	Mix	No	Damaged	Discard; or use it for alternative purposes	Repair and
4	9	8 × “Size 1”, 1 × “Size 3”	“Size 1” top, “Size 3” bottom	No	Damaged	Repair and continue to use	Repair and continue to use
5	2	1 × “Size 2”, 1 × “Size 4”	“Size 4” roof, “Size 2” bottom	No	Damaged	Repair and continue to use	Repair and continue to use
6	2	1 × “Size 2”, 1 × “Size 4”	“Size 4” roof, “Size 2” bottom	Partial (Size 4)	Damaged	Repair and continue to use	Repair and continue to use
7	25	22 × “Size 1”, 1 × “Size 2”, 2 × “Size 3”	Mix	No	Damaged	Repair and continue to use; Discard; or use it for alternative purposes	Repair and continue to use

^a^Hole size categories based on the WHO guidelines [10]: “Size 1”: smaller than a thumb (0.5–2 cm), “Size 2”: larger than a thumb but smaller than a fist (2–10 cm), “Size 3”: larger than a fist but smaller than a head (10–25 cm) and “Size 4”: larger than a head (> 25 cm)

^b^Each side panel split into top half and bottom half

^c^Type of repair: Sewing with needle and thread (as per SNP BCC messaging)

^d^Physical damage categories based on total hole surface area [10]: good: < 79 cm^2^, Damaged: 80–789 cm^2^ and Too Torn: > 790 cm^2^

To compare reported intentions during the PA with actual behaviour, the net used by the person being interviewed was assessed onsite at the end of each IDI. The number, size and location of holes and evidence of repair were recorded, and participants were asked to reflect on the status of their nets and their reported attitudes to care and repair. The holes were assessed using the World Health Organization (WHO) hole size descriptions and categorized as either “good” (< 79 cm^2^ hole surface area), “damaged” (80–789 cm^2^) or “too torn” (> 790 cm^2^) [10].

### Data management and analysis

All audio-recorded data from the FGDs and IDIs were transcribed and spot-checked by both the interviewer and note-taker involved in the interview. Following approval of transcripts, interview summaries were written for each FGD and IDI. Data analysis was conducted following thematic framework analysis procedures [36] to specifically explore study objectives. The thematic framework analysis included familiarization of data, identification of the thematic framework, indexing, charting, mapping and interpretation [37–40]. An initial coding framework was created using the topic guide. All four researchers who participated in the data collection then independently conducted an inductive thematic analysis of the interview summaries and a preliminary coding framework was established including sub-themes relevant to study objectives. Names and all individual identifiers were removed from transcripts.

The transcripts were then entered into NVivo 11 Pro software (QSR International Pty Ltd, Australia) for final data management, indexing, and identification of associated narratives to the study objectives. Data collected were organised by coding responses under each theme identified in the final codes to allow within and between participant group analysis. Data from the structured questionnaires was summarized. Triangulation was done to compare (a) responses given during the PA, (b) observations made in the mosquito net assessment, and (c) participant reflections of their current net status to provide in-depth context and to validate findings.

## Results

A total of 118 individuals from the two villages were interviewed (male: n = 56; female: n = 62). Fifty-eight people were from the village of Makanjiro (rural) and 60 from the village of Kilimahewa (peri-urban). The highest level of education attained by the majority of the study participants (n = 87) was completion of primary school. Ninety-one participants reported to have received their LLIN from the SNP while 27 nets were purchased from local stores. There are no data on whether shop-bought nets were treated or untreated. Eighty-six of the 263 children of the study participants were attending primary school and therefore eligible for a mosquito net through the SNP. On average, the study participants received 0.5 SNP nets per year. Of the 118 interviewed participants, 87 had been exposed to BCC about malaria in the past 6 months. The most recalled BCC messages were to hang the net, sleep underneath the net and use the net all year round.

### Perceived threat

Malaria was unanimously perceived to be a major public health threat in Ruangwa. The disease was mainly associated with death, miscarriage and poverty. Illness forced individuals to be away from the workforce while malaria treatment increased household costs. The disease was reported to weaken the bodies of those who suffered from it, and the repercussions would be worse if the head of household fell ill as reiterated by a woman in Kilimahewa.

“*Yes, I am unable to perform any of my tasks because I am sick. I am unable to care for my children or work. If the father, who is the head of household, falls sick, it is even worse as there is no*-*one to provide*.” (IDI participant, Woman with child under the age of five, Kilimahewa)

Generally, the importance of mosquito nets for protection against malaria mosquitoes was reported as the main driver of motivation to care and repair nets by the majority of the study participants.

*“The net protects me so that a mosquito who would bite and infect me with malaria cannot reach me.”* (IDI participant, Man, Kilimahewa)

Participants reported a high risk of being bitten by mosquitoes and valued the protection of the nets from mosquitoes which aided better sleep.

“*For the poor, sleep is leisure. If you hear noises from such insects, you will not sleep*.” (FGD Participant, Makanjiro, Older man)

Mosquito nets used by children, especially those under the age of five, were most likely to be repaired first. This was because young children were reported to be most vulnerable to the disease and not able to care for or repair their own nets. Male key informants and older men reported their own personal nets to be of top priority for repair as they were the breadwinners of the family. Older women specifically reported to repair damaged household items, including nets and clothes, in one sitting rather than repairing each item soon after each hole was identified.

Nails on bed frame edges were reported as the primary cause of damage because of the daily tucking and untucking from underneath the mattress. Other causes of damage included children playing with the net, pulling the net too much to fit a bed that is bigger than the net, edges of the wooden frame “besela” used to hang the net, and household pests and rodents.

### Net care

Net care was primarily defined as washing, tying up the net over the sleeping space in the morning and lowering it in the evening for use, and seasonal storage. Upon probing, hanging nets after washing and drying nets inside or outside the household were acknowledged as other practices associated with care.

Nets were usually washed within the household compound in a basin or bucket with soap and water as soon as the net was perceived to be dirty. Most participants reported washing their nets every other week. Washing the net ensured cleanliness, which also prevented other dirt-related problems such as sneezing and headaches. Tying up nets over the sleeping space in the morning and lowering it in the evening for use was done to avoid mosquitoes and other insects from hiding inside the net during the day. Seasonal storage, a result of seasonal net use, differed between the two villages. Kilimahewa (peri-urban) residents reported using mosquito nets throughout the year whereas Makanjiro (rural) residents only used their nets during the rainy season when mosquito prevalence increased, except for households with children under the age of 5.

“*We use mosquito nets during rainy season, because there are a lot of swamps and mosquitoes, but during the dry season, there are no mosquitoes. We store the nets.”* (FGD participant, Older Woman. Makanjiro)

When describing barriers to net care or repair, study participants were quick to separate themselves from the subject and started speaking in the third person. Reported barriers to net care included care not being a priority in the day-to-day activities, “negligence” and lack of net “Ngao” (net retreatment kits that used to be sold over the counter but were discontinued in 2009 after the introduction of LLINs). Women attributed being pre-occupied by other household activities such as sweeping and cooking, which left them too exhausted by the end of the day to then take particular care of the net. It was also reported difficult to keep up with small children who would play and tug on the nets if tied above the sleeping space.

“*Other people do not have time to relax at home because they are so preoccupied by other household activities that they even forget to tie up nets in the morning.”* (IDI Participant, Woman, Kilimahewa).

The majority of participants reported that other community members were negligent as they did not clean or care for their personal items. These community members were not expected to make time to wash or care for the nets provided to them. There were concerns that nets needed to be re-treated with insecticides after each wash to activate the insecticide for continued protection as was previously recommended with “*Ngao*” net retreatment kits. The lack of net retreatment kits at the markets left many heads of households in dilemma of how frequently to wash their nets.

*“For most residents here, our households are of dirt floors, so when you sweep the house, in no time your net is dirty.”* (FGD Participant, Older Men, Kilimahewa).

Key informants reported poverty as the underlying barrier to net care. The general household environment such as mud floors and grass/thatch roofs makes it difficult to care for one’s net every day. Resources such as a wooden frame “besela” required to hang up the net during the day were also not available for all.

*“For many it is about their general standard of living. It is not only difficult to care for their nets but also for other household items such as clothes.”* (FGD Participant, Young Man, Kilimahewa)

### Net repair

**Fig. 3:**
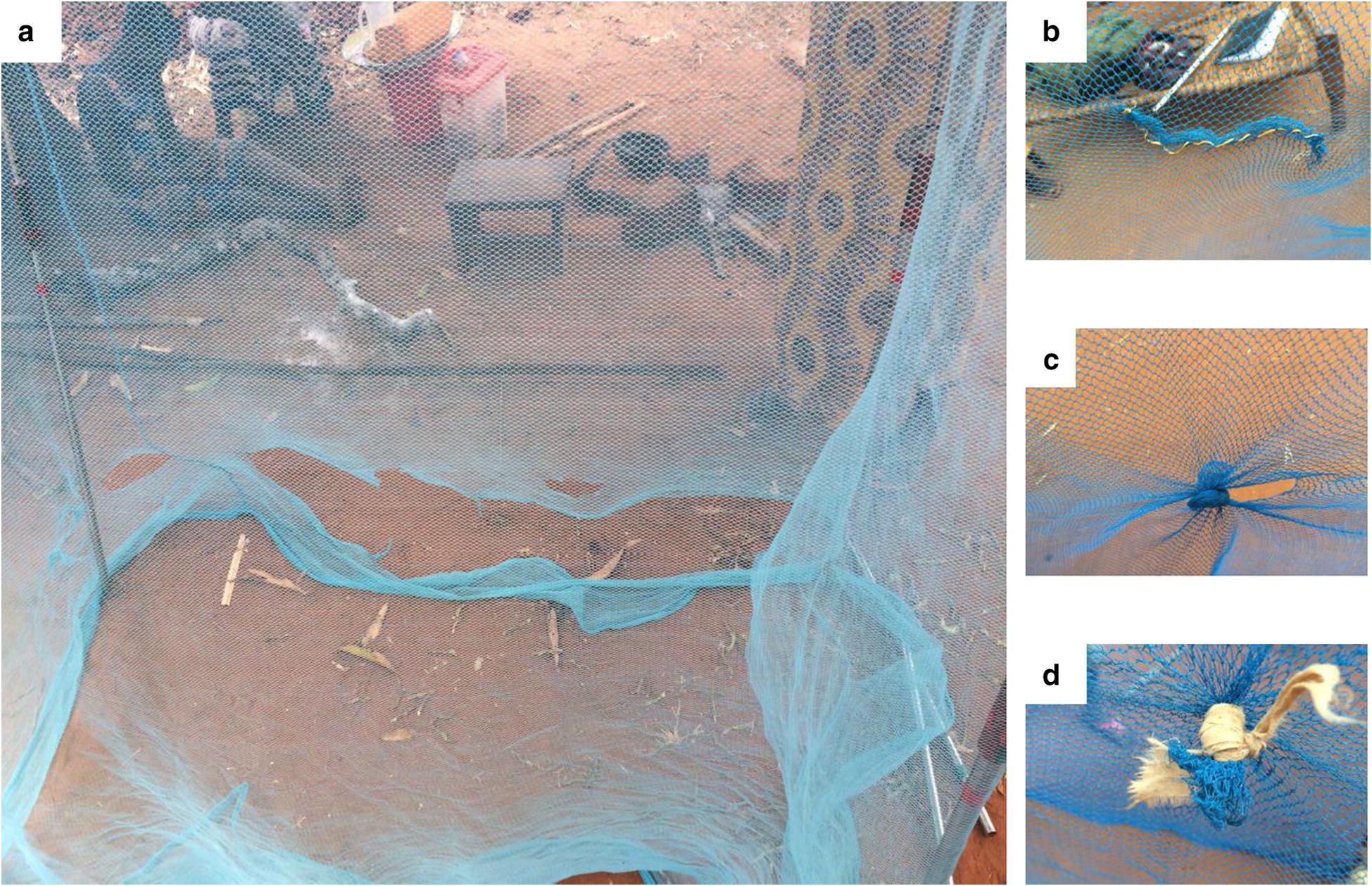
Mosquito net assessment. **a** An illustration of the mosquito net assessment on a collapsible frame outside the household; **b** net repair by sewing; **c** partial net repair by tying a knot and **d** complete net repair by tying a knot

Net repair was reported necessary as soon a hole was identified and defined as either sewing and/or tying knots (Fig. 3). Upon probing, adding patches to holes was dismissed as an option for net repair. Though patches of old clothes were easy to find, sewing them on the net reduced the airflow inside the net, and was hence not seen as a practical solution for repair.

Blocking the entry of mosquitoes into the net was crucial, because,

“*if mosquitoes enter the net because I do not repair it, the children will get malaria and I will have to stop doing everything else to take care of them and maybe even get malaria myself*.” (IDI participant, Woman with child under the age of five, Makanjiro)

Study participants generally echoed their huge dependence on freely-distributed nets as the primary source and means of protection against malaria. While nets were available at the local shops, the costs were perceived too high even for untreated nets (approximately TZS 10,000, USD$4.50). Replacement schedules of the free SNP nets were largely unclear to residents in the study villages so extending the life of a net until a replacement net arrives, free or bought, was reported crucial to ensure household members remain protected for as long as possible.

Net repair was perceived a social responsibility for all LLIN recipients. Through net repair, community members, who are the workforce to build the Tanzanian nation, would be protected from the deadly disease of malaria.

“*When we join forces and work together, we create a workforce that a village such as ours depends on for development. But when community members fall sick with malaria, we lose the workforce in the village, and also as a nation.”* (FGD participant, Key Informant, Makanjiro)

Net repair was largely reported as a temporary measure before the acquisition of a new net, hence not a matter of priority. Some participants reported sewing a net as too much work, while others reported not knowing how to sew a net given the varying material type and mesh size of the net itself. The lack of educational sessions on when to repair nets was also reported as a barrier. When holes were not repaired, the number and size of holes increased until nets were perceived to be “too torn” to be worth repairing.

“*Some do not know what to do when they identify a hole on the net. Some do not even recognize that the hole should be repaired to adequately protect themselves from malaria.*” (IDI participant, Woman, Kilimahewa)

Mechanistic problems reported included regular needles being too small to grip properly and close the hole whereas tying a knot was only feasible for some types of holes (Fig. 3). Lack of self-initiative to explore and find alternative solutions, for example using bigger needle and thicker thread to repair the net, was reported as a potential barrier for others to repair their nets. Some participants also reported lack of sewing kits (needle and thread) for net repair readily available in their households. Key informants highlighted that some tailors refused to mend nets as nets were perceived as too personal to be repaired by them.

### Self-efficacy to care for and repair nets

Both men and women reported their capabilities to perform all the basic care and repair practices such as washing, hanging, tying up the net above sleeping space, storing it away and knotting. However, the wife or woman was seen as the one solely responsible for net care and repair in households irrespective of her economic role (i.e. whether she was head of household or also worked). The man’s main contributions were to act as the catalyst (proposing when care actions such as washing should be performed) and the financial decision-maker (net repair and/or replacement decisions). In the absence of a woman (unmarried, widowed or travelling wife), men reported to care for and repair their own nets but in the confines of their household in seclusion from the public. Children aged 13 and above, irrespective of their gender, could take responsibility of their own nets. Parental check-up became less common due to cultural norms that refrain mothers from entering their sons’ room and the father a daughter’s room once the children reached puberty.

### Mosquito net assessment

Of the nets presented during the Participatory Activity (PA), the following five net IDs from the PA; 1, 3, 4, 5 and 7 (Table 1), were most comparable to those from participating households in terms of level of damage and lack of repair (Table 2). All participants reported they *would* repair the single hole identified at the bottom of net ID 1. The horizontal tear was perceived easy to sew together if sewing materials were readily available in their households. Alternatively, participants suggested that the hole located at the bottom could be tucked under the mattress. Most study participants reported they would discard Net ID 3 (18 holes) or use it for alternative purposes around the household such as an additional cushion under the mattress or fencing the flower garden. The holes were perceived to be too many and too scattered to repair. As with Net ID 1, the hole located at the bottom of Net ID 4 (9 holes) was reported to be either repaired or tucked underneath because “*it* [the single hole] *is located at the bottom. After tucking the net under the mattress, mosquitoes cannot get through*.” (IDI participant, Older Man, Kilimahewa).

**Table 2:** Mosquito net assessment findings by In-Depth Interview participant groups and village

Village/participant group	Net type	Number of holes	Hole sizes^a^	Hole location^b^	Repair^c^	Category^d^
Kilimahewa (peri-urban)						
Man	LLIN	3	3 × “Size 1”	Bottom	0	Good
Man	LLIN	3	3 × “Size 1”	Bottom	0	Good
Man	LLIN	36	17 × “Size 1”, 19 × “Size 2”	Top, bottom, roof	5	Damaged
Man	Unknown	31	16 × “Size 1”, 14 × “Size 2”, 1 × “Size 3”	Top, bottom	1	Damaged
Man	Untreated	3	3 × “Size 1”	Bottom	0	Good
Woman	LLIN	9	8 × “Size 1”, 1 × “Size 2”	Top, bottom	0	Good
Woman	LLIN	1	1 × “Size 2”	Bottom	0	Good
Woman	LLIN	1	1 × “Size 1”	Top	0	Good
Woman	LLIN	7	6 × “Size 1”, 1 × “Size 2”	Bottom	2	Good
Woman	LLIN	21	19 × “Size 1”, 2 × “Size 2”	Top, bottom	0	Damaged
Woman with under 5	Unknown	106	98 × “Size 1”, 8 × “Size 2”	Top, bottom, roof	2	Damaged
Woman with under 5	Untreated	4	2 × “Size 1”, 2 × “Size 2”	Bottom	0	Good
Woman with under 5	LLIN	2	2 × “Size 1”	Top, bottom	0	Good
Woman with under 5	LLIN	13	2 × “Size 1”, 10 × “Size 2”, 1 × “Size 3”	Bottom	0	Damaged
Woman with under 5	LLIN	4	1 × “Size 1”, 3 × “Size 2”	Top, bottom	2	Damaged
Makanjiro (rural)						
Man	LLIN	4	2 × “Size 2”, 1 × “Size 3”, 1 × “Size 4”	Top, bottom	0	Too torn
Man	LLIN	12	12 × “Size 1”	Bottom	0	Good
Man	LLIN	21	8 × “Size 1”, 13 × “Size 2”	Bottom	5	Damaged
Man	LLIN	2	2 × “Size 1”	Bottom	1	Good
Man	LLIN	4	1 × “Size 1”, 1 × “Size 2”, 2 × “Size 3”	Top, bottom	0	Damaged
Woman	LLIN	0	–		0	Good
Woman	LLIN	0	–		0	Good

^a^Hole size categories based on the WHO guidelines [10]: “Size 1”: smaller than a thumb (0.5–2 cm), “Size 2”: larger than a thumb but smaller than a fist (2–10 cm), “Size 3”: larger than a fist but smaller than a head (10–25 cm) and “Size 4”: larger than a head (> 25 cm)

^b^Each side panel split into top half and bottom half

^c^Number of holes repaired on the net. Type of repair varied as per Fig. 3 including sewing and knotting

^d^Physical damage categories based on total hole surface area [10]: Good: < 79 cm^2^, Damaged: 80–789 cm^2^ and Too Torn: > 790 cm^2^

Very few of the small holes located at the top were noticed by participants, and those that did identify them, did not mention any action to repair them. Participants responded they would either repair and continue to use net ID 5 (2 holes) or use it for alternative purposes around the house depending on their financial status when the holes were identified. A few reported they would seek out the local tailor to repair the large hole at the top. Reponses for net ID 7 (25 holes) were mixed with some ready to use it for alternative purposes while others would repair and continue to use it. However, it was unanimously echoed that all the nets presented in the PA were still usable and *should* be repaired as the holes were not overwhelming in number or size. Study participants did not perceive any of the nets presented to be too torn; therefore, they *should* all be repaired for continued use of protection against malaria, particularly when left with no money to acquire a new net (Table 1).

Generally, mosquito nets assessed in peri-urban Kilimahewa were in “good” condition (n = 10) while the remaining handful of nets (n = 5) were “damaged” as per WHO hole sizes categories [10] (Table 2). The condition of nets assessed in Makanjiro varied much more: Two nets were in as good as new condition (no holes), four nets had some holes but were still in “good” condition, five nets were “damaged”, and four nets were “too torn” (Table 2). Of the 30 nets assessed across the two villages, only five nets in Kilimahewa and three nets in Makanjiro showed any evidence of repair by sewing or knotting.

The most common response during the PA was to repair and continue to use nets, and everyone reported they should repair and continue to use. However, actual evidence of repair in nets from households was scarce (Table 2). When asked, the main reasons given for not repairing nets were; (1) not being able to identify most of the holes while inside the households due to poor lighting, and (2) tucking holes located at the bottom underneath the mattress. Study participants did indicate that the net assessment exercise encouraged them to repair the holes in their nets and that they would assess all other nets present in their households for damage following the end of the interview.

### Cues to action

Given that the SNP was the primary source of nets in the study villages, it was suggested that parents should be invited to the schools for educational sessions on net care and repair so that they could engage better daily in the maintenance of LLINs to prevent malaria.

It was proposed that Community Health Workers and other experts from the district headquarters should train people on the importance of nets, how to care for nets and when to repair them. However, there were some participants that cautioned:

“*Mosquito nets are private items that one has to have self*-*initiative to take care of. Educational sessions on such sensitive matters can be deemed offensive by the recipient of the net*” (FGD participant, Older Man, Makanjiro).

The women generally echoed that men were equally as capable to perform both care and repair duties within households, hence should also participate in day-to-day activities. Net manufacturing companies were requested to produce stronger nets. It was also requested that net retreatment kits “*Ngao*” should be restocked in the commercial markets as it was reassuring to retreat a net after each wash to ensure it would repel or kill mosquitoes upon contact.

Upon probing, mass washing sessions, inclusion of leaflets and sewing kits in the packaging, and road shows were perceived as other measures to encourage net maintenance and general cleanliness. However, it was emphasized that the leader of the mass washing initiative should be someone not associated with the village to avoid passing judgement and spreading gossip of the status of nets within the village.

Information on leaflets attached on the packaging of nets was received with mixed reviews. While those in Kilimahewa received it well, study participants in Makanjiro worried for the illiterate who were perceived to be the majority in the village despite previous distributions including leaflets with pictorial demonstrations. Interactive educational sessions by community health workers and experts during road shows were proposed to be more informational.

## Discussion

Though not unanimously actioned, there was a general readiness to care for and repair mosquito nets in southern Tanzania for the love of a good night’s sleep free of mosquito bites or noises, as observed in other studies across sub-Sahara Africa [5, 12–14]. Response saturation was reached quickly in our study among participant groups and between villages, and responses of motivators and perceived challenges were similar to those of other studies in sub-Saharan Africa. This implies that general motivators and barriers to net care and repair are comparable across a range of cultural settings. These results are discussed using the theoretical framework presented in Fig. 1 and based on the HBM [23]. This study found that malaria was perceived to be a major threat and that mosquito nets were considered a useful tool against mosquito bites and to reduce health expenses associated with disease (individual perceptions; Fig. 2). Most people felt they were able to take good care of their nets and repair them when necessary (self-efficacy), although net repair was most commonly seen as a temporary measure and net care was performed mainly to keep nets clean and free of insects rather than to specifically prolong the lifespan of the net (potential barriers). A discrepancy was found between what people reported they did or knew they should do and actual condition of the nets. This highlights potential gaps in knowledge and uncovers the lack of an important motivator to care and repair: the better the net condition, the better the protection against malaria (likelihood of action).

Study participants much preferred net care over repair, which was similar to studies in West and East Africa [5, 12–14]. In southern Tanzania, the motivation for net care was generally associated with overall net maintenance such as cleanliness and preventing mosquitoes and other insects from hiding inside the net, and not directly associated with the prevention of damage as in other studies. Similarly to other studies, however, dirty nets were perceived harmful to one’s health and shameful to society [5, 13–15]. Clean nets were seen as aesthetically pleasing and a show of a responsible woman. Some net owners reported to wash their nets almost every other week (approximately 26 washes a year) as was also observed in Uganda [5] and Peru [41]. Tanzania’s School Net Programme BCC messaging currently lacks a recommendation for washing frequency and only states to “*wash your net when it gets dirty and dry it in the shade to preserve the effectiveness of the insecticide of the net*” (Pamela Kweka [John Hopkins Centre for Communications Programs in Tanzania] *pers. comm*.). The existing BCC also does not address the fact that LLINs do not require the “Ngao” net retreatment kits. Households were left in a dilemma as they wanted clean nets, yet also wanted to maintain the active chemical content. If they did not wash the nets, they got negative reactions from family members. If they did wash their nets frequently, the nets were deemed ineffective to sleep under after about a year. In Kenya, increased washing frequency was associated with decreased physical condition of nets [8]. In Tanzania, 45% of nets were in bad condition after washing them four to seven times a year and insecticidal content was also observed to be low [42].

Behavioural Change Communication should be updated to include a realistic recommendation regarding washing frequency as was done in Peru [41], keeping in mind that expecting people to refrain from washing their subjectively dirty nets is unrealistic [15]. Behaviour Change Communication should also highlight the importance of preventing damage on nets while promoting preventative net maintenance behaviours, such as tying up the net over the sleeping space or storing nets safely away from children or rodents when not in use [23].

Although participants stated that nets were important to protect against malaria, net repair was only seen as a temporary measure before acquisition of a new net as was also found in Senegal [13, 43]. People much preferred receiving brand-new nets for free and only uncertainty around distribution schedules motivated net repair. Although people reported that net repair was necessary as soon as a small hole was identified, inconsistencies were observed between such reported intentions and the physical condition of nets observed inside households [5, 14]. The lack of priority to repair nets led to the accumulation of holes with time. Nets observed to be “too torn” showed no more evidence of repair and were from households of women (self-reported primary caretakers) (Table 2). Households with poor lighting, which were the majority in the study villages, have more difficulty in identifying holes for repair. Using a frame, which stretched the material as was done in this study, allowed participants to easily identify the smallest of holes. This, however, is an unlikely method for household members to regularly assess their own nets so they can determine the appropriate action. When the net is removed from its hanging place, it is normally crumpled together in a ball of fabric, making it difficult to identify small holes. Many larger holes were observed at the bottom of the nets and respondents most often said they would tuck those holes underneath the mattress. The convenience of tucking holes underneath the mattress fostered neglect for other holes. Thus, holes that could not be tucked underneath the mattress were stretched and became larger over time.

Mechanistic challenges may have contributed to the low occurrence of repairs. Net repairs by sewing was largely dependent on other household items requiring sewing, was time consuming and needed financial investment of a bigger needle and thicker thread (Fig. 3b). Alternatively, knotting was either partial or pulled a lot of net material together depending on the size of the hole, potentially creating other mosquito entry points (Fig. 3c, d). In Nigeria, net repairs were not sufficient to improve overall status, i.e. shift nets from the “damaged” to the “good” WHO category [25], irrespective of the increase in proportion of repairs on torn nets [44].

Lack of knowledge or misconceptions (e.g. Ngao) were identified as key barriers to effective care and repair practices. Existing SNP BCC primarily targeted primary and secondary school children through posters and a weekly radio programme called “Pata Pata” jingle. Children were advised to inform their parents or caretakers of care and repair practices. Subsequently this may have created a knowledge gap where some parents and caretakers received limited or diluted information from their children. Workshops engaging parents, who have primary responsibility of taking care of the nets, were requested. Behavioural Change Communication for SNP should build on existing practices around the villages to share public health information of the developments of malaria control interventions such as the transition from use of untreated nets, retreatments kits and now LLINs [45] to ensure appropriate continued community-wide engagement in net maintenance. Women of Makanjiro village reported increased motivation to care for their LLINs following a Community Change Agent’s educational session in their small group “Vikoba” meetings. Community-wide engagements in Ghana [46], Cambodia [47] and Madagascar [48] have had positive effects on promoting interactions with malaria control interventions and should become a more regular feature as part of continuous net distribution mechanisms in Tanzania.

The BCC messages that were recalled by household members emphasize the proper use of LLINs. It is therefore important to evolve the BCC strategy to include positive social norms, e.g. the personal responsibility to maintain nets in good condition [5, 13], especially as the SNP is now embedded into the NMCP LLIN strategy and has expanded its distribution to the Lake Zone [27]. Messages should incorporate net care as part of a daily routine and not as an additional burden to ensure that the luxury from a good night’s sleep and health gains are maintained.

### Study limitations

Though sampling was continued even after response saturation was reached, these findings only reflect the attitudes and actions of those interviewed and not the entire Lindi region or other zones in Tanzania where residents with school-going children continuously receive nets from the SNP. Although the researchers explained they were not health workers or involved in the SNP distribution process, there remains a possibility that study participants missed the distinctions, potentially biasing responses to be favourable towards mosquito nets and reported care and repair behaviours. The mosquito net assessment and PA were done outside the house on a frame that stretched the netting in a way that even the smallest holes could be identified. The study did not follow-up to assess whether any of the nets observed with damage were repaired as per study participant claims, and how they were repaired.

## Conclusion

There was willingness to both care and repair mosquito nets in Ruangwa district, although net care was more likely to be performed than repair. Promotion of care practices as means to prevent net damage including realistic recommendations for washing frequency need to be included in the BCC messaging to prevent over-washing of nets. Discrepancies were observed between reported intentions to repair mosquito nets and current net condition which further reinforces the findings of previous studies that demonstrated no substantial benefit to promoting net repair. Targeted education through health facilities, particularly workshops for parents and engagement with community change agents were recommended as potential means to overcome barriers to net care by the study community.

## Abbreviations

BCC: Behavioural-Change Communication
FGDs: Focus Group Discussions
HBM: Health Belief Model
IDI: In-Depth Interview
IHI: Ifakara Health Institute
LLINs: long-lasting insecticidal net
NMCP: National Malaria Control Programme
PA: participatory activity
PMI: U.S President’s Malaria Initiative
SAVVY: Sample Vital registration with Verbal Autopsy
SNP: School Net Programme
SPD: Sentinel Panel of Districts
TNVS: Tanzania National Voucher Scheme
WHO: World Health Organization
